# Near-infrared photoimmunotherapy with galactosyl serum albumin in a model of diffuse peritoneal disseminated ovarian cancer

**DOI:** 10.18632/oncotarget.12710

**Published:** 2016-10-17

**Authors:** Toshiko Harada, Yuko Nakamura, Kazuhide Sato, Tadanobu Nagaya, Shuhei Okuyama, Fusa Ogata, Peter L. Choyke, Hisataka Kobayashi

**Affiliations:** ^1^ Molecular Imaging Program, Center for Cancer Research, National Cancer Institute, NIH, Bethesda, Maryland, 20892, USA

**Keywords:** near-infrared photoimmunotherapy, ovarian cancer, peritoneal cancer metastases, galactosyl serum albumin, beta-D-galactose receptor

## Abstract

Near-infrared photoimmunotherapy (NIR-PIT) is a highly cell-selective cancer therapy based on an armed antibody conjugated with a phthalocyanine-based photo-absorber, IRDye700DX (IR700). NIR-PIT can quickly kill target cells that express specific proteins on the cellular membrane but only when the antibody-IR700 conjugate binds to the cell membrane and is then exposed to NIR light. NIR-PIT is highly selective based on the specificity of the antibody. Galactosyl serum albumin (GSA) is composed of albumin decorated with galactose molecules conjugated to the carboxyl groups of albumin. GSA binds to beta-D-galactose receptors, a surface lectin, which are overexpressed on the cell surface of many cancers, including ovarian cancers and is quickly internalized after binding. Here, we demonstrate the feasibility of NIR-PIT in a model of disseminated peritoneal ovarian cancer (SHIN3 cells) using GSA-IR700 that binds to beta-D-galactose receptors. GSA-IR700 bound quickly to SHIN3 cells, then accumulated in the endo-lysosomes. Cell-specific killing was observed *in vitro*, yet a relatively large dose of NIR light exposure was required for cell killing compared to antibody-IR700 conjugates. To evaluate *in vivo* therapeutic effects of GSA-IR700 NIR-PIT, peritoneal disseminated SHIN3 tumor-bearing mice were separated into four groups: no treatment; NIR light only; GSA-IR700 only; and GSA-IR700 NIR-PIT. Repeated NIR-PIT showed significant suppression of tumor based on bioluminescence compared to the other groups (p < 0.05). Thus, repeated NIR-PIT using GSA-IR700 can achieve efficient antitumor effects, although GSA-IR700 NIR-PIT was less effective than antibody-IR700 NIR-PIT conjugates likely due to the rapid internalization of GSA-IR700.

## INTRODUCTION

Epithelial ovarian carcinoma (EOC) is the leading cause of death among patients with gynecologic malignancies. 85% of women with EOC present with advanced stage III or IV disease, leading to high morbidity and mortality. Cytoreductive surgery followed by systemic chemotherapy is the standard therapy for advanced-stage ovarian cancer. Overall five-year survival (OFS) rate of all ovarian cancer patients has been improved by 2.0 % each year from 2003 to 2012, reaching 46 % in 2015. However, the OFS rate of patients with advanced ovarian cancer remains at only 28 % [1, 2]. Removal of peritoneal metastases as small as 1 mm or even less in diameter has been demonstrated to improve the OFS of patients [3, 4]. However, poorly differentiated ovarian cancers often lead to diffuse peritoneal dissemination consisting of a large number of sub-millimeter lesions which are unresectable. Thus, even after cyto-reductive surgery, recurrence of disease is likely.

Near-infrared photoimmunotherapy (NIR-PIT) is a recently developed, target cell-specific cancer treatment that involves an antibody-photoabsorber conjugate (APC) that binds to the cellular membrane of target cells and is subsequently exposed to NIR light [5]. A typical APC consists of a cancer-specific monoclonal antibody (mAb) and a photo-absorber, IRDye700DX (IR700), which is a silica-phthalocyanine derivative that can be covalently conjugated to the antibody [5]. The APC binds to an antigen on the cellular membrane. Irradiation with NIR light at 690 nm induces immediate necrotic (immunogenic) cell death primarily by causing damage to the cell membrane in a cell-specific manner. Previous *in vitro* studies demonstrated that NIR-PIT is highly cell-selective, so that non-targeted cells immediately adjacent to the targeted cells in co-cultures are left undamaged [5]. A variety of different antibodies, which recognize different target antigens have been successfully employed as NIR-PIT APCs. Several distinct types of antibodies including chimeric, humanized and total human antibodies have also been successfully employed NIR-PIT agents [6–12].

However, non-antibody mediated NIR-PIT has proven more difficult. There are several potential advantages of using non-antibody targeting agents including cost and availability. In this study, we used galactosyl serum albumin (GSA) as the targeting moiety for an NIR-PIT agent. GSA is comprised of galactose molecules conjugated via carboxyl groups to an albumin molecule, and this construct binds weakly to beta-D-galactose receptors [13, 14]. The beta-D-galactose receptor is an H-type lectin that is overexpressed on the surface of various cancer cells, including ovarian cancers [15–18]. When GSA is bound to D-galactose receptors, it is quickly internalized and accumulated in the endo-lysosome. Therefore, GSA is mostly found in the endo-lysosome and minimally observed on the cell surface, a potential disadvantage for NIR-PIT, which primarily thought to affect the cell membrane [13–17]. In addition, upon intraperitoneal (i.p.) administration, unbound GSA conjugate is absorbed rapidly through the peritoneum and trapped by D-galactose receptors on hepatocytes [14, 15, 17], resulting in high target-to-background ratios in the peritoneal cavity but the potential for off-target effects in the liver. However, a major advantage of GSA as a target is that it appears ubiquitous among tested ovarian cancers whereas it is difficult to find a single antibody that will bind to all types of ovarian cancers. Thus, D-galactose receptor-targeted therapy for peritoneal metastases could be advantageous for the treatment of ovarian cancer, if the disadvantages of rapid endo-lysosomal uptake can be overcome.

In this study, we investigated GSA-IR700 as a candidate NIR-PIT agent in an animal model of the human ovarian cancer cell line, SHIN3, which overexpresses D-galactose receptor and produces diffuse peritoneal dissemination [13].

## RESULTS

### *In vitro* characterization of GSA-IR700

**Figure 1:**
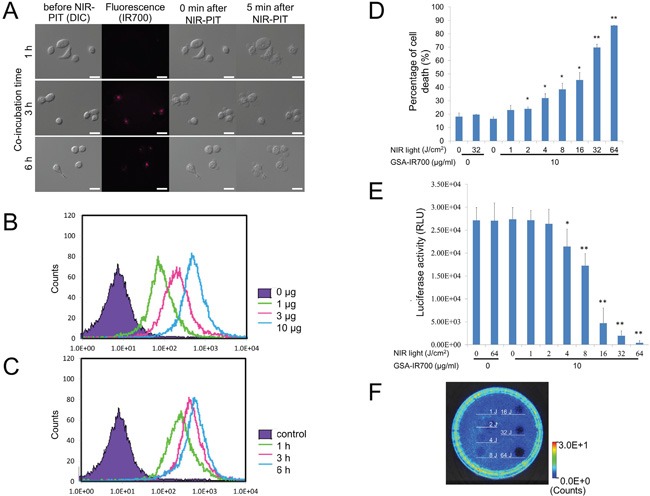
**A.** Differential interference contrast (DIC) and fluorescence microscopy images of SHIN3 cells after incubation with GSA-IR700 for 1, 3, and 6 h. The small fluorescent dots were seen in the cytoplasm 3 and 6 h after incubation. Necrotic cell death was observed upon excitation with NIR light (after 0, 5 min) regardless of incubation time. Scale bars = 20 μm. **B.** Dose and **C.** time-course examination using flow cytometric analysis. Binding affinity of GSA-IR700 to SHIN3 cells was examined. GSA-IR700 showed dose-dependent binding affinity to SHIN3 cells. GSA-IR700 bound to SHIN3 cells by 1 h of incubation, with increasing up to 3 h then plateaued. **D.** Membrane damage induced by NIR-PIT was measured by a dead cell count using SYTOX Green staining, which increased in a NIR light dose dependent manner (n = 3, *p < 0.05, **p < 0.01 vs. untreated control, by Student's t-test). **E.** Luciferase activity in SHIN3-luc cells was measured in relative light units (RLU), which decreased in a NIR light dose dependent manner (n = 6, *p < 0.05, **p < 0.01 vs. untreated control, by Student's t-test) **F.** Bioluminescence imaging (BLI) of a 10 cm dish. Luciferase activity in SHIN3-luc cells decreased in a NIR light dose dependent manner.

Fluorescence microscopic images of SHIN3 cells showed low fluorescence 1 h post-incubation (Figure 1A). At 3 and 6 h post-incubation, GSA-IR700 bound to SHIN3 cells could be identified as small fluorescent dots in the cytoplasm that is typical of uptake within endo-lysosomes, but surface binding was minimal.

The fluorescence intensity of GSA-IR700 bound to SHIN3 cells increased in a concentration dependent manner (Figure 1B). There was increased accumulation of GSA-IR700 within SHIN3 cells up to 3 h then it plateaued (Figure 1C).

### *In vitro* NIR-PIT

Immediately after exposure, NIR light induced cellular swelling, bleb formation, and rupture of vesicles typical of NIR-PIT responses *in vitro*. This has previously been shown to represent necrotic cell death and occurred at all incubation times. Most of these morphologic changes were observed within 5 min of light exposure, indicating rapid induction of necrotic cell death (Figure 1A).

Based on the incorporation of SYTOX Green, the percentage of cell death increased in a light dose dependent manner (Figure 1D). No significant cytotoxity was observed with NIR light alone in the absence of GSA-IR700 or with GSA-IR700 alone without NIR light. However, in GSA-IR700 cells exposed to NIR light, cytotoxity was observed albeit at a relatively lower intensity compared to the previous studies using the APC trastuzumab-IR700 against another ovarian cancer cell line (SKOV3) [12]. Bioluminescence imaging (BLI) showed a decrease in luciferase activity in a light dose-dependent manner (Figure 1E), comparable to the result obtained by SYTOX Green staining. BLI of a 10 cm dish also demonstrated that luciferase activity in SHIN3-luc cells decreased in NIR-PIT treated cells but not in controls (Figure 1F).

### *Ex vivo* evaluation of tumor specific accumulation

**Figure 2:**
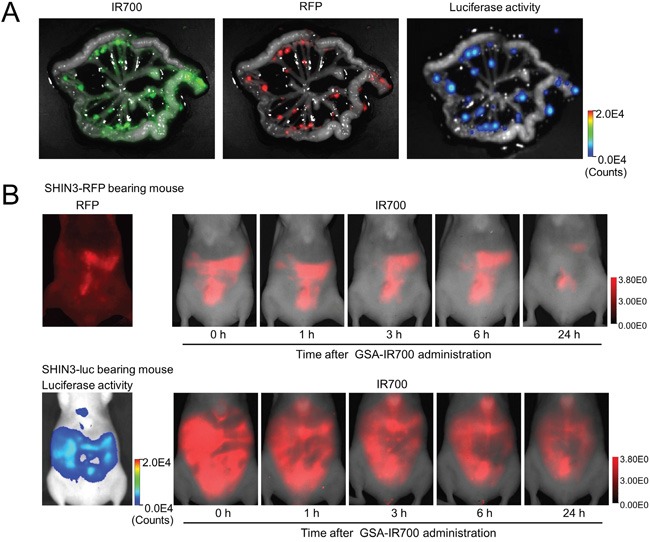
**A.** IR700, RFP fluorescence, and luciferase activity image of extracted mesentery derived from a SHIN3-luc-RFP tumor bearing mouse 3 h after i.p. administration of GSA-IR700. The fluorescence of IR700 was confirmed to be mostly coincident with RFP positive foci. Moreover, the fluorescence of luciferase activity was also confirmed to be mostly coincident with RFP positive foci. **B.** *In vivo* serial IR700 fluorescence images of SHIN3-RFP (upper row) and SHIN3-luc (lower row) using tumor-bearing mice with administration of GSA-IR700. The distribution of GSA-IR700 correlated well with the fluorescence of RFP or luciferase activity without evident accumulation in other organs up to 6 h after GSA-IR700 administration.

The fluorescence of IR700 coincided with red fluorescence protein (RFP) positive foci. Moreover, bioluminescence derived from luciferase activity also coincided with RFP positive foci, indicating that bioluminescence accurately depicted cancer location. In addition, the IR700 fluorescence signal showed tumor specific accumulation with high tumor-to-background ratio (TBR) (Figure 2A).

### GSA-IR700 distribution assay

The distribution of GSA-IR700 correlated well with RFP and luciferase activity without evident accumulation in other organs up to 6 h after GSA-IR700 administration (Figure 2B). Most of the fluorescence derived from GSA-IR700 was washed out by 24 h after administration. In addition, BLI was more sensitive for tumors and exhibited lower background signal compared to RFP fluorescence. Based on this result, *in vivo* NIR-PIT was performed 3 hours after GSA-IR700 administration.

### *In vivo* NIR-PIT

**Figure 3:**
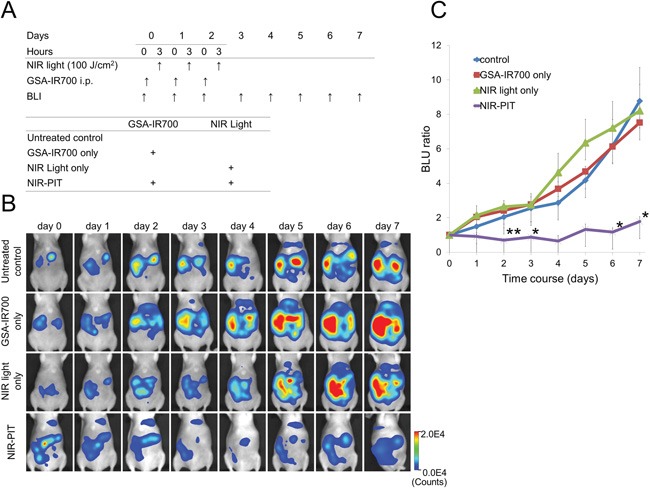
**A.** Outline of *in vivo* NIR-PIT regimen. **B.** BLI of SHIN3-luc tumor bearing mice was obtained every day up to day 7. Luciferase activity was decreased only in the NIR-PIT group. **C.** Quantitative analysis of RLU ratio. Significant suppression of increment of RLU ratio was seen in the NIR-PIT group compared to other groups (n = 5, *p < 0.05, **p < 0.01 vs. other groups, Tukey's HSD test with ANOVA).

Treatment effect was quantified as relative light unit (RLU) ratio, a ratio of total RLUs compared to day 0 (before treatment) (Figure 3B and 3C). The RLU ratio was significantly inhibited in the NIR-PIT treated group (p < 0.01, 0.044, 0.049 and 0.042 for day 2, 3, 6 and 7, respectively). No significant therapeutic effect was observed in the control groups including those receiving GSA-IR700 i.p. only or NIR light only.

## DISCUSSION

NIR-PIT is a recently developed, target-cell-selective cancer treatment that occurs after administration of an antibody-IR700 conjugate is allowed to bind to target cells which are then exposed to NIR light [5]. While NIR-PIT has demonstrated efficacy against a variety of tumors, it appears to result from cell membrane-mediated damage and thus, is optimized for antibody-antigen cell surface binding [6–12]. In this study, we investigated GSA-IR700 as an alternative to antibody conjugates for NIR-PIT in a peritoneal disseminated ovarian cancer model overexpressing the beta-D-galactose receptor. The result of the *in vitro* study using SHIN3 cells showed accumulation of GSA-IR700 mostly in the endo-lysosome of the cancer cells. The cytotoxic effect of *in vitro* NIR-PIT was light dose dependent but less effective than NIR-PIT employing APCs such as trastuzumab-IR700 targeting HER2-expressing ovarian cancer (SKOV3) [12]. The cell-killing effect of NIR-PIT is maximized when the APC binds to cell surfaces rather than when APC is internalized [5]. However, because GSA loosely binds to beta-D-galactose receptor and then is quickly internalized into cells [13], it is predicted to be a less effective NIR-PIT agent. What was uncertain was whether it could be sufficiently effective to be a useful treatment in the absence of a suitable antibody. Thus, *in vitro* NIR-PIT with GSA-IR700 showed a weak therapeutic effect due to loose binding of GSA-IR700 to the cell surface and massive internalization of GSA-IR700 into cells. This result tends to confirm that NIR-PIT is a cell surface membrane-mediated event and that it is optimized when the targeting moiety remains bound to the cell surface.

The result of *ex vivo* and *in vivo* evaluation showed tumor-specific accumulation of the IR700 fluorescence signal with high TBR, indicating that GSA-IR700 is a potential agent for visualizing ovarian cancer. GSA-expressing tumors can be potentially treated with NIR-PIT although more light is needed compared to APCs. Since GSA is rapidly absorbed from the peritoneum, intraperitoneally injected GSA-IR700 typically only binds cells within 0.2 mm depth from the surface [19]. Repeated regimens of NIR-PIT improves the efficacy of GSA-IR700 delivery deeper into tumor nodules because when GSA-IR700 is administered i.p., the first treatment kills the surface layer of the tumor, peels off, then subsequently administered GSA-IR700 can penetrate more deeply into the tumor. Therefore, we conducted *in vivo* NIR-PIT three times every 24 hours. Significant inhibition of the RLU ratio was seen in the NIR-PIT treated group (p < 0.01). However, the decrease of RLU ratio after NIR-PIT was not observed consistently over the entire time course unlike conventional antibody-mediated NIR-PIT [12]. However, it also should be noted that growth inhibition was observed on the fast-growing SHIN3 peritoneal dissemination model in this study, while SKOV3 peritoneal dissemination model treated with trastuzumab-IR700 showed relatively slow growth and therefore, may be more sensitive to NIR-PIT effects [12].

This study has several limitations. In small mice, NIR light could be delivered into the peritoneal cavity only with surface illumination, yet this could not in humans. In order to deliver NIR light into the human peritoneal cavity, direct exposure of NIR light to the peritoneal cavity would be necessary using either an open abdomen or light applied via laparoscopes. Applying NIR light to the treatment of residual tumor while tissues are still exposed after surgical resection might be a way to incorporate NIR-PIT into the management of patients. Laparoscopic approaches using fiber-coupled laser diodes with diffuser tips could also be effective. Another limitation is that our model was only evaluated in immunocompromised mice. The immunological reaction to NIR-PIT should be evaluated in the future in immunocompetent mice where it is anticipated that significant immunogenic cell death will occur. In addition, we used RFP as a fluorescent protein for monitoring tumor growth and found a little less sensitive than bioluminescence imaging mostly due to high autofluorescence background in red spectra in the mouse abdomen. Therefore, green or near infrared emitting fluorescent proteins can surely monitor tumors better than RFP [20–24].

In conclusion, GSA-IR700 demonstrated binding to the beta-D-galactose-positive tumor cell line, SHIN3, and then rapidly internalized within tumor cells. Subsequent NIR-PIT showed target-specific necrotic cell death both *in vitro* and moderate therapeutic effects *in vivo*. Tumor growth suppression was induced by a repeated regimen of NIR-PIT in a peritoneal disseminated model. Although GSA-IR700 NIR-PIT appears to be less effective than comparable antibody-mediated NIR-PIT, it could nevertheless have an impact for treating various types of beta-D-galactose receptor over-expressing tumors, a common phenotype among ovarian cancers. This may be especially useful when specific antibodies are not available for ovarian cancers. The development of similar non-antibody-based forms of NIR-PIT could therefore be an effective way of extending the utility of this novel therapy to other tumors.

## MATERIALS AND METHODS

### Reagents

Water soluble, silica-phthalocyanine derivative IRDye700DX NHS ester (IR700; C_74_H_96_N_12_Na_4_O_27_S_6_Si_3_, molecular weight of 1954.22) was obtained from LI-COR Bioscience (Lincoln, NE, USA). GSA, which has 23 galactosamine residues conjugated to bovine serum albumin (BSA), was purchased from Sigma Chemical (St. Louis, MO, USA). All other chemicals used were of reagent grade.

### Synthesis of IR700-conjugated GSA

At room temperature, GSA (1 mg, 14 nmol) was incubated with IR700 (273.9 μg, 140 nmol, 10 mmol/L in DMSO) and 0.1 mol/L Na_2_HPO_4_ (pH 8.5) at room temperature for 2 h. The mixture was purified with a gel filtration column (Sephadex G 25 column, PD-10, GE Healthcare, Piscataway, NJ, USA), and the GSA-binding fraction (2.6–3.8 mL) was eluted by 0.066 M phosphate-buffered saline (PBS) at pH 7.4 and collected in a test tube. We abbreviate IR700 conjugated to GSA as GSA-IR700. GSA-IR700 was kept at 4°C in the refrigerator as a stock solution. The protein concentrations of GSA-IR700 samples were determined using the Coomassie Plus protein assay kit (Pierce Biotechnology, Rockford, IL, USA) by measuring the absorption at 595 nm with a UV-Vis system (8453 Value UV-Visible Value System; Agilent Technologies, Santa Clara, CA, USA) using standard solutions of known concentrations of GSA (100 μg/mL). The concentration of IR700 was measured by absorption at 689 nm with the UV-Vis system to confirm the number of fluorophore molecules conjugated to each GSA molecule. The number of fluorophore molecules per GSA was adjusted to be approximately five.

### Cell culture and transfection

An established ovarian cancer cell line, SHIN3, was used in this study. A luciferase stably expressed SHIN3 cell line was also established with a transfection of RediFect Red-FLuc-Puromycin Lentiviral Particles (PerkinElmer, Waltham, MA, USA). High luciferase expression was confirmed with 10 passages. A red fluorescence protein (RFP) stably expressed SHIN3 cell line was also established by transfection with RFP (EF1a)-Neo lentiviral particles (AMSBIO, Cambridge, MA, USA). High RFP expression was confirmed in the absence of a selection agent after 10 passages. A luciferase and RFP stably expressed SHIN3 cell line was also established by transfection with RediFect Red-FLuc-Puromycin Lentiviral Particles and RFP (EF1a)-Neo lentiviral particles. High luciferase expression and high RFP expression were confirmed in the absence of a selection agent after 10 passages. We abbreviate these cell lines as SHIN3-luc, SHIN3-RFP, and SHIN3-luc-RFP, respectively. All cells were grown in RPMI 1640 (Invitrogen, Gaithersburg, MD, USA) supplemented with 10 % Fetal Bovine Serum (FBS; Invitrogen), 0.03 % L-glutamine and 1 % Penicillin/streptomycin (Life Technologies) in tissue culture flasks and maintained in a humidified incubator at 37°C in a 5 % carbon dioxide environment.

### Fluorescence microscopy

Two thousand SHIN3 cells were seeded onto covered, glass-bottomed 35 mm cell-culture dishes and pre-incubated for 16 h. Then, GSA-IR700 was added to the medium at 10 μg/mL, and the cells were incubated for 1, 3, and 6 h at 37°C in a humidified incubator. The medium was then removed and replaced with fresh culture medium. Fluorescence microscopy was performed (Olympus BX 61 microscope; Olympus America Inc., Melville, NY, USA) with the following filters: excitation wavelength 590–650 nm, since IR700 has a sub-absorption peak at 600–650 nm; and emission wavelength 665–740 nm. Transmitted light differential interference contrast (DIC) images were also acquired.

### Flow cytometry

To examine the appropriate dose, one hundred thousand SHIN3 cells were seeded into a 24-chamber culture well and pre-incubated for 16 h. After replacing the medium with fresh culture medium, GSA-IR700 was added to the medium at 1, 3, and 10 μg/mL, and the cells were incubated for 6 h at 37°C in a humidified incubator. To examine the appropriate timing, one hundred thousand SHIN3 cells were seeded into a 24-chamber culture well and pre-incubated for 16 h. After replacing the medium with fresh culture medium containing 10 μg/mL GSA-IR700, the cells were incubated for 1, 3, and 6 h at 37°C in a humidified incubator. An argon-ion laser (488 nm) was used for excitation. Signals from cells were collected with a 653- to 669 nm band-pass filter for GSA-IR700. Cells were analyzed in a FACS Calibur (BD, BioSciences, San Jose, CA, USA) and CellQuest software (BD BioSciences).

### *In vitro* NIR-PIT

The cytotoxic effects of NIR-PIT were determined by flow cytometric SYTOX Green (Thermo Fisher Scientific Inc., Waltham, MA, USA) staining and bioluminescence imaging (BLI). For SYTOX Green staining, one hundred thousand SHIN3 cells were seeded into a 24-chamber culture well and pre-incubated for 16 h. After replacing the medium with fresh culture medium containing 10 μg/mL GSA-IR700, the cells were incubated for 3 h at 37°C in a humidified incubator. After washing with PBS, phenol-red-free culture medium was added. Then, cells were exposed with a NIR laser, which emits light at 685–695 nm wavelength (BWF5-690-8600-0.37; B&W TEK INC., Newark, DE, USA). The actual power density (mW/cm^2^) was measured using an optical power meter (PM 100, Thorlabs, Newton, NJ, USA). One hour after treatment, cells were re-suspended with PBS containing SYTOX Green (final 2 μg/mL) and were then incubated at room temperature for 15 minutes in the dark, followed by flow cytometry. Signals from SYTOX Green were collected using a 515- to 545 nm band-pass filter.

For BLI, either two hundred thousand SHIN3-luc cells were seeded into a 24-chamber culture well or twenty million cells were seeded onto a 10 cm dish; both were pre-incubated for 16 h. After replacing the medium with fresh culture medium containing 10 μg/mL GSA-IR700, the cells were incubated for 3 h at 37°C in a humidified incubator. After washing with PBS, phenol-red-free culture medium was added. Then, cells were exposed with a NIR laser, and D-luciferin (150 μg / ml in PBS per well; Gold Biotechnology, St. Louis, MO, USA) was administered to PBS-washed cells 1 h after NIR-PIT. Luciferase activity was assayed using a BLI system (Photon Imager; Biospace Lab, Paris, France), with results reported in relative light units (RLU). Regions of interest (ROI) were placed on each entire well, and the sum of RLU was then calculated using M^3^ Vision Software (Biospace Lab).

### Animal model

All procedures were performed in compliance with the Guide for the Care and Use of Laboratory Animal Resources (1996) by the National Research Council and were approved by the local Animal Care and Use Committee. SHIN3-RFP, SHIN3-luc, and SHIN3-luc-RFP cells (2×10^6^ cells each) were i.p. administered to female, homozygote, athymic nude mice (6–8 weeks of age, National Cancer Institute Animal Production Facility, Frederick, MD, USA). During the procedure, mice were anesthetized with 1.5 % isoflurane.

### *Ex vivo* evaluation of tumor specific accumulation of GSA-IR700

SHIN3-RFP cells can be used for cancer location especially for *ex vivo* imaging [25]. However, fluorescence of RFP is often limited in *in vivo* applications and bioluminescence is more reliable for *in vivo* assessment of treatment. Thus, we conducted *ex vivo* evaluation using SHIN3-luc-RFP cells to validate whether the location of luciferase activity coincided with that of RFP. Five days after SHIN3-luc-RFP cell administration, GSA-IR700 (25 μg / 300 μL in PBS per mouse) was i.p. administered to SHIN3-luc-RFP tumor-bearing mice. Three hours after GSA-IR700 administration, mice were euthanized by carbon dioxide inhalation. Immediately after euthanasia, the abdominal walls were excised and the small-bowel mesentery was extracted and spread on a nonfluorescent plate. First, D-luciferin (3 mg / 200 μL per specimen) was sprayed on the mesentery and luciferase activity was evaluated five minutes later using a BLI system. Next, spectral fluorescence images were acquired continuously using the Maestro *in vivo* imaging system (CRi, Waltham, MA, USA). The following filter sets were used: for RFP fluorescence imaging, excitation 575–605 nm band-pass filter and emission 645 nm long-pass filter; for IR700 fluorescence imaging, excitation 615–665 nm band-pass filter and emission 700 nm long-pass filter. The tunable emission filter was automatically stepped in 10 nm increments from 600 to 800 nm at constant exposure to generate a spectral image for both RFP and IR700 imaging. The spectral fluorescence images from RFP and IR700 were then unmixed using commercial software (Maestro software; Cri, Waltham, MA, USA).

### *In vivo* distribution assay after i.p. administration of GSA-IR700

*In vivo* distribution after i.p. administration of GSA-IR700 was performed using SHIN3-RFP and SHIN3-luc cells to verify whether the location of fluorescence from GSA-IR700 coincided with SHIN3-RFP or SHIN3-luc tumor *in vivo*. Five days after cell administration, GSA-IR700 (25 μg/ 300 μL in PBS per mouse) was i.p. administered. Fluorescence images of IR700 were obtained using a Pearl Imager (LI-COR Biosciences) with a 700 nm fluorescence channel at 0, 1, 3, 6, and 24 h after administration.

For SHIN3-RFP-bearing mice, spectral fluorescence images of RFP were acquired before administering GSA-IR700 using the Maestro *in vivo* imaging system with an excitation 575–605 nm band-pass filter and an emission 645 nm long-pass filter. The tunable emission filter was automatically stepped in 10 nm increments from 600 to 800 nm at constant exposure to generate a spectral image. The spectral fluorescence images include the autofluorescence spectra, and the spectra from RFP which were then unmixed based on their spectral patterns.

For SHIN3-luc-bearing mice, D-luciferin (3 mg / 200 μL in PBS per mouse) was i.p. administered and luciferase activity was assayed using a BLI system.

### *In vivo* NIR-PIT

SHIN3-luc cells were used for *in vivo* NIR-PIT study because the treatment effect could be evaluated by BLI. Five days after SHIN3-luc cell administration, SHIN3-luc tumor-bearing mice were randomly allocated into the following four treatment groups of five mice per group: (i) no treatment (control); (ii) NIR light exposure only at 100 J/cm^2^ (500 mW/cm^2^ * 200 s) (NIR light only); (iii) GSA-IR700 (25 μg / 300 μL in PBS per mouse) i.p., no NIR light exposure (GSA-IR700 only); and (iv) GSA-IR700 i.p. administration (25 μg / 300 μL in PBS per mouse), NIR light was administered at 100 J/cm^2^ (500 mW/cm^2^ * 200 s) 3 h after GSA-IR700 i.p. (NIR-PIT). These therapies were performed every day up to 3 days (Figure 3A).

The abdomen of mice were externally exposed with NIR light from the ventral side of the mouse while the upper abdominal area was shielded from light exposure by covering with an aluminum foil to prevent light exposure to liver. BLI was performed five minutes after i.p. administration of D-luciferin (3 mg / 200 μL in PBS per mouse) once each day, from day 0 to day 7. ROIs were placed over the entire abdomen except the shielded upper abdominal area, and the sum of the RLU was calculated.

### Statistical analysis

Quantitative data were expressed as mean ± s.e.m. (standard error of the mean) from a minimum of three experiments, unless otherwise indicated. Statistical analysis was performed using GraphPad Prism (GraphPad Software, La Jolla, CA, USA). Unpaired t-tests were used to compare differences between two groups. For multiple comparisons, a one-way analysis of variance (ANOVA) followed by Tukey's honestly significant difference (HSD) test was used. P values less than 0.05 were considered to be statistically significant.
